# Effects of Underwater Noise Exposure on Early Development in Zebrafish

**DOI:** 10.3390/ani15152310

**Published:** 2025-08-07

**Authors:** Tong Zhou, Yuchi Duan, Ya Li, Wei Yang, Qiliang Chen

**Affiliations:** 1 Chongqing Key Laboratory of Conservation and Utilization of Freshwater Fishes, Animal Biology Key Laboratory of Chongqing Education Commission of China, Chongqing Normal University, Chongqing 401331, China; a19923011672@163.com (T.Z.); duanyuchi2000@163.com (Y.D.); yali010708@163.com (Y.L.); 2 National Engineering Research Center for Inland Waterway Regulation, Chongqing Key Laboratory of Ecological Waterway, Chongqing Jiaotong University, Chongqing 400044, China; cqjtuyw@cqjtu.edu.cn

**Keywords:** noise pollution, embryonic development, oxidative damage, zebrafish, temporal patterns

## Abstract

Anthropogenic noise pollution is a significant global environmental issue that adversely affects the behavior, physiology, and auditory functions of aquatic species. However, studies on the effects of underwater noise on early developmental stages of fish remain scarce, particularly regarding the differential impacts of daytime versus nighttime noise exposure. In this study, zebrafish (*Danio rerio*) embryos were exposed to control group (no additional noise), daytime noise (100–1000 Hz, 130 dB, from 08:00 to 20:00) or nighttime noise (100–1000 Hz, 130 dB, from 20:00 to 08:00) for 5 days, and their embryonic development and oxidative stress levels were analyzed. Compared to the control group, the results indicated that exposure to both daytime and nighttime noise led to delays in embryo hatching time and a significant decrease in larval heart rate. Notably, exposure to nighttime noise significantly increased the larval deformity rate. Noise exposure, particularly at night, elevated the activities of catalase (CAT) and glutathione peroxidase (GPX), as well as the concentration of malondialdehyde (MDA), accompanied by upregulation of antioxidant-related gene expression levels. Nighttime noise exposure significantly increased the abnormality rate of otolith development in larvae and markedly downregulated the expression levels of *otop1* related to otolith development regulation, while daytime noise exposure only induced a slight increase in the otolith abnormality rate. After noise exposure, the number of lateral neuromasts in larvae decreased slightly, yet genes (*slc17a8* and *capgb*) related to hair cell development were significantly upregulated. Overall, this study demonstrates that both daytime and nighttime noise can induce oxidative stress and impair embryonic development of zebrafish, with nighttime noise causing more severe damage.

## Simple Summary

Underwater noise pollution poses a growing threat to fish populations, yet its developmental impacts on fish remain poorly understood. As a well-established model organism in environmental and developmental research, zebrafish (*Danio rerio*) provides an ideal model to investigate these effects. This study examined the effects of daytime and nighttime underwater noise exposure on zebrafish using an embryo model. The results showed that noise exposure delayed embryo hatching and reduced larval heart rates, with nighttime noise causing more severe developmental issues. Furthermore, noise exposure induced oxidative stress, impaired otolith development, and altered the expression of hair cell-related genes. These findings underscore the significance of noise pollution timing and offer valuable insights for implementing time-specific noise control measures to protect aquatic biodiversity.

## 1. Introduction

Noise, defined as any unwanted or disturbing sound, pervades both terrestrial and aquatic ecosystems. Recognized as the second greatest environmental health risk after air pollution, noise exposure has been linked to numerous adverse health effects including cardiovascular diseases, sleep disorders, and impaired cognitive development [1,2], leading to its characterization as an “invisible killer” in public health discourse. In the underwater environment, noise levels have been rising steadily due to urban development, the expansion of shipping and transportation networks, underwater resource extraction, and seismic exploration [3,4]. For instance, underwater noise in the Northeast Pacific has increased by 12 dB over 40 years [5]. Anthropogenic noise, particularly low-frequency noise (<1000 Hz), overlaps with the auditory sensitivity range of most fish, masking critical acoustic signals and disrupting their survival [6]. Anthropogenic noise pollution is also a serious threat to freshwater ecosystems. Freshwater environments are generally shallower and more enclosed than open seas, producing distinct sound propagation characteristics that increase exposure risk for aquatic organisms [7,8].

Anthropogenic underwater noise, such as ship navigation and wading construction (e.g., river bank dredging, bridge pile foundation construction in shallow water area and dam maintenance engineering, etc.), may cause hearing loss, developmental abnormalities, and behavioral disorders in aquatic species [6]. Previous studies have shown that underwater noise can increase the concentration of plasma cortisol in fish [9,10,11]. While reactive oxygen species (ROS) serve as essential signaling molecules in physiological processes, their excessive generation induces oxidative stress through disruption of redox homeostasis [12]. Anthropogenic underwater noise pollution has been demonstrated to significantly impair the antioxidant defense systems in aquatic organisms, as evidenced by dysregulation of key enzymatic components [11]. Underwater noise can also alter oxygen consumption and respiratory metabolic rates of fish [13,14,15], affect intestinal flora and reduce their immunity [16,17]. High-intensity noise can also induce temporary or permanent hearing threshold shifts in fish. For example, the hearing threshold of fathead minnow (*Pimephales promelas*) increased after 2 h of exposure to white noise (300–4000 Hz, 142 dB), but fully recovered within 6 days; however, after 24 h of noise exposure, the hearing threshold did not return to the baseline even after 14 days [18]. Hearing loss resulting from threshold shifts is often associated with damage to sensory hair cells [19]. When pink snappers (*Pagrus auratus*) were exposed to airgun noise, the sensory epithelium of the inner ear suffered extensive damage, manifested as ablated hair cells [20]. Moreover, noise can frighten fish and disrupt their behavior, leading to changes in activities such as schooling, foraging, and offspring care [21,22,23,24,25]. Studies on zebrafish (*Danio rerio*) have demonstrated noise-induced modifications in swimming patterns, foraging behavior, and auditory sensitivity [26,27,28]. However, these studies predominantly focused on juvenile and adult fish and largely overlooked the effects of noise on the early life stages (embryo–larva) of fish.

Early life stages are critical for fish development and population dynamics. During this period, fish are highly vulnerable to environmental stressors due to their limited mobility and ongoing physiological development. Zebrafish is an ideal model for such studies due to their transparent embryos and well-characterized development [29]. Studies have shown that zebrafish can sense sounds in the frequency range of about 300 Hz to 2000 Hz, especially in the low-frequency region (<1000 Hz), the sensitivity peak is approximately at 800 Hz [30,31,32,33,34]. This auditory ability is present in both juvenile and adult zebrafish stages, making it an ideal model for studying the effects of acoustic exposure. Additionally, zebrafish is an important model for sleep research, being a diurnal animal that is active during the day and rests at night [35]. In this study, zebrafish embryos were exposed to daytime noise (100–1000 Hz, 130 dB, from 08:00 to 20:00) or nighttime noise (100–1000 Hz, 130 dB, from 20:00 to 08:00) for 5 days in order to compare the effects of daytime and nighttime noise on the embryonic development of zebrafish. The findings of this study can provide valuable references for underwater noise pollution management and fish breeding protection.

## 2. Materials and Methods

### 2.1. Subjects and Housing Conditions

Adult zebrafish (AB strain) were obtained from the National Zebrafish Resource Center (Wuhan, China). All adult zebrafish were adaptively reared for 2 weeks in a recirculating aquaculture system before the experiment with no deaths during this period (28 ± 0.5 °C, pH 7.4 ± 0.2, dissolved oxygen ≥ 6 mg/L) under a 12:12 h light–dark cycle. Adult zebrafish were fed commercial feed (Shandong Shengsuo Feed Technology Co., Ltd., Yantai, China) twice daily at 09:00 and 17:00. To ensure water quality stability, the aquaculture system was replenished with aerated, dechlorinated rearing water every two days.

Fertilized zebrafish embryos at 2 h post fertilization were obtained from adult fish according to Chen et al. [36] and were randomly distributed into 9 tanks (50 × 40 × 30 cm; containing 50 L water), with 300 embryos each tank. There were three experimental groups (three tanks per group): (1) a control group (no additional noise), (2) a daytime noise exposure group (100–1000 Hz, 130 dB, from 08:00 to 20:00), and (3) a nighttime noise exposure group (100–1000 Hz, 130 dB, from 20:00 to 08:00). All procedures were approved by the Animal Care and Use Committee of Key Laboratory of Animal Biology of Chongqing at Chongqing Normal University (approval No. Zhao-20231012-02).

### 2.2. Experimental Design and Noise Exposure Protocol

**Figure 1:**
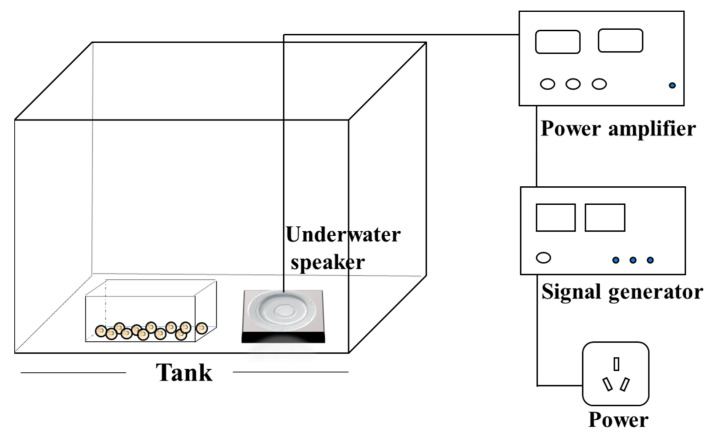
Schematic diagram of the experimental setup for underwater noise exposure.

The noise exposure equipment was similar to that in our recent study [37] and is shown in Figure 1. Noise was generated using a signal generator (DG1022Z, RIGOL, Beijing, China), amplified by a power amplifier and then output to the tank (50 cm × 40 cm × 30 cm) via an underwater speaker (UW-30, Electro-Voice, Inc., Burnsville, MN, USA) to simulate the underwater noise environment [38]. Sound pressure levels in noise exposure tanks were measured and calibrated using a digital hydrophone (VST-DH series, Nanjing Haohai Ocean Technology Co., Ltd., Nanjing, China) with MATLAB-based (R2023b) frequency analysis to ensure a consistent intensity of 130 dB within the 100–1000 Hz range (the underwater reference sound pressure is 1 μPa). The underwater ambient noise level in the control group was around 86 dB re 1 μPa. All tanks were placed on polystyrene foam platforms and surrounded by soundproofing material to minimize external acoustic interference. The noise parameters (100–1000 Hz, 130 dB) were selected to realistically simulate anthropogenic underwater noise, as this frequency range encompasses both the predominant spectrum of human-generated noise and the auditory sensitivity of most fish species, while the intensity represents ecologically relevant exposure levels documented in field measurements [39,40,41].

The noise treatment lasted for 5 days under controlled conditions (28 ± 0.5 °C, pH 7.4 ± 0.2, dissolved oxygen ≥ 6 mg/L) with a 12:12 light–dark cycle under 600 lux daytime illumination. During this period, zebrafish embryos underwent critical development (e.g., organogenesis), enabling detection of noise impacts on survival, development, and physiological responses [38].

During the experimental process, the hatching rate and mortality were recorded every 24 h. Embryonic development into larvae began at approximately 48 h post-fertilization and was completed by approximately 96 h post-fertilization. Dead embryos or larvae were promptly removed. At 72 h, 10 larvae were randomly sampled per tank for otolith development assessment and heart rate measurement (1 min counts under an optical microscope (Chongqing Aopu Optoelectronic Technology Co., Ltd., Chongqing, China, UY2031)). Concurrently, 30 larvae from each tank were homogenized in 1 mL ice-cold RNAiso Plus (Cat#: 9109, TaKaRa, Dalian, China) reagent and stored at −80 °C for subsequent real-time fluorescence quantitative (RT-qPCR) analysis. At 120 h, the deformity rate was calculated (malformed larvae/total survivors ×100%) and representative malformations were documented photographically. Additional samples were collected for lateral neuromast hair cell staining (10 larvae/tank) and RT-qPCR (20 larvae/tank), with remaining specimens being flash-frozen in liquid nitrogen and stored at −80 °C for biochemical assays.

### 2.3. Biochemical Assays

Larvae samples were homogenized in ice-cold physiological saline (1:9 *w*/*v*) using a tissue homogenizer, followed by centrifugation at 3000× *g* for 15 min at 4 °C. The resulting supernatant was carefully collected and analyzed for superoxide dismutase (SOD, Cat#: A001-3-1), catalase (CAT, Cat#: A007-1-1), and glutathione peroxidase (GPX, Cat#: A005-1-2) activities, as well as malondialdehyde (MDA, Cat#: A003-1-1) concentrations using commercial kits (Nanjing Jiancheng Bioengineering Institute, Nanjing, China) following the manufacturer’s instructions, to assess oxidative stress levels [42].

### 2.4. Hair Cell Staining and Lateral Neuromast Counting

Zebrafish larvae were immersed in a 1 μmol/L YO-PRO-1 solution (Cat#: C2022, Beyotime Biotechnology, Shanghai, China), incubated in feeding water for 1 h in the dark, and then washed with water [43]. The larvae were anesthetized with MS-222 (0.01% *w*/*v*) for 1–2 min and placed under a stereo fluorescence microscope (Olympus IX73, Olympus Corporation, Tokyo, Japan) for observation and photographing, and the number of posterior lateral neuromasts was counted.

### 2.5. RT-qPCR

Analyses of gene transcript levels were conducted by RT-qPCR, following the MIQE guidelines [44] as described by He et al. [37]. Twenty or thirty larvae were combined and used as a single biological sample for total RNA extraction using RNAiso Plus. RNA concentration was quantified using a Nanodrop ND-2000 spectrophotometer (Thermo Electrom Corporation, Waltham, MA, USA). RNA purity and integrity were evaluated based on two criteria: (1) absorbance ratios (A260/280 = 1.8–2.1; A260/230 > 2.0) and (2) visualization of intact 18S and 28S ribosomal RNA bands via 1% agarose gel electrophoresis. Only RNA samples meeting both quality control standards were processed for downstream analysis. The PrimeScript™ RT reagent Kit with gDNA Eraser (Cat#: RR047A, TaKaRa, Dalian, China) was used for reverse transcription. Gene expression levels were quantified by use of TB Green^®^ Premix EX Taq™ Ⅱ (Cat#: RR820A, TaKaRa, Dalian, China). The primer sequences used in this study are presented in Table 1. The cycling parameters were as follows: 95 °C for 30 s, followed by 40 cycles of 95 °C for 5 s, 60 °C for 30 s, and 72 °C for 30 s. Finally, a melting curve analysis was performed. Standard curves generated for each target gene using serially diluted cDNA templates demonstrated comparable amplification efficiencies ranging from 95% to 105%, meeting the recommended criteria for RT-qPCR analysis. Two housekeeping genes (*ef1α* and *β-actin*) were used to normalize the transcriptional expression levels of target genes, and the detection results were calculated using the 2^−ΔΔCt^ method [45].

### 2.6. Statistical Analysis

The data were analyzed using SPSS 26.0 software (IBM, Armonk, NY, USA). Data normality was first assessed with the Shapiro–Wilk test, and independent *t*-tests were performed to compare groups, with the Holm–Bonferroni correction applied for comparisons. The results are presented as mean ± standard error of the mean (SEM), with statistical significance set at *p* < 0.05. The figures were generated using GraphPad Prism 8 (GraphPad Software, San Diego, CA, USA; https://www.graphpad.com/scientific-software/prism/, accessed on 26 July 2025).

## 3. Results

### 3.1. Development Parameters

**Figure 2:**
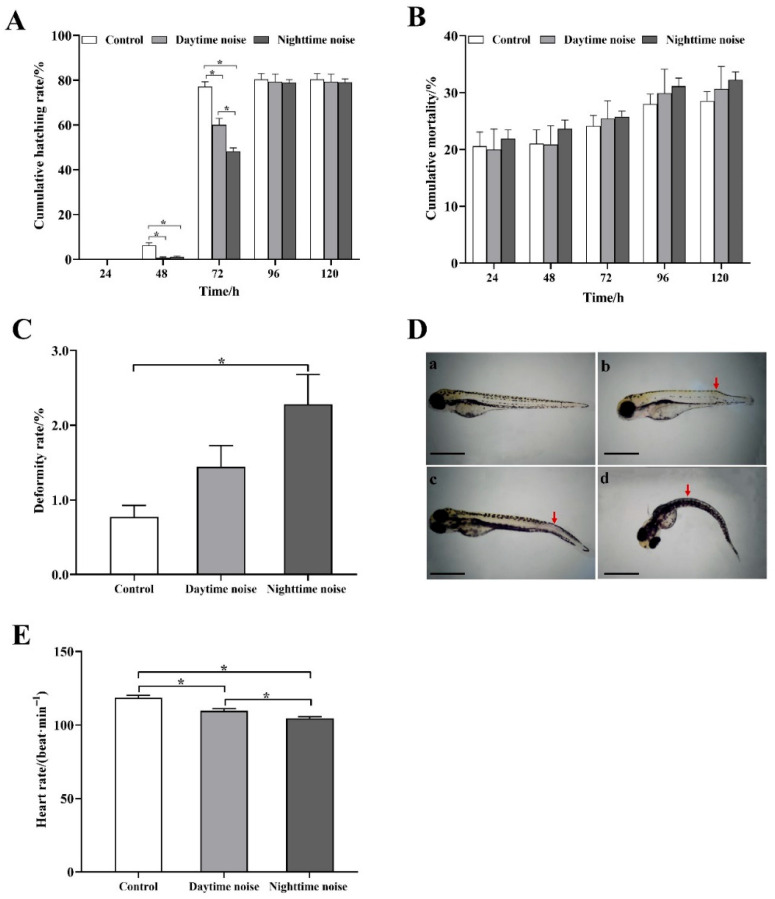
Developmental impacts of daytime and nighttime noise exposure on zebrafish embryos and larvae. (**A**) Cumulative hatching rate; (**B**) cumulative mortality rate; (**C**) larval deformity rate; (**D**) representative images of normal and malformed larvae with arrows indicating deformities ((**a**) normal larvae; (**b**–**d**) malformed larvae; scale bar = 1 mm); (**E**) heart rate. The data represent mean ± SEM (*n* = 3 replicates for (**A**–**C**), *n* = 30 for (**E**)). The asterisks (*) denote significant differences between groups (*p* < 0.05); the comparisons between groups without asterisk annotations indicate no significant differences (*p* ≥ 0.05).

Although the final cumulative hatching rate at 120 h showed no significant differences among groups, both daytime and nighttime noise exposure significantly delayed embryo hatching (Figure 2A). At 48 h, the cumulative hatching rates in daytime (*t* (4) = 4.8, *p* = 0.026) and nighttime (*t* (4) = 4.52, *p* = 0.026) noise exposure groups were significantly lower than in controls, and at 72 h, the cumulative hatching rates in daytime (*t* (4) = 4.59, *p* = 0.020) and nighttime (*t* (4) = 10.42, *p* < 0.001) noise exposure groups were also significantly lower than in controls, with nighttime exposure showing more pronounced effects. While the groups showed no significant differences in mortality rates (Figure 2B), the noise-exposed groups had a consistently higher mortality from 72 to 120 h compared to controls, particularly the nighttime noise group. Compared with the control group, the larval deformity rate increased following both daytime and nighttime noise exposure, with a statistically significant elevation observed in the nighttime noise group (*t* (4) = −3.524, *p* = 0.048; Figure 2C). Additionally, compared with the control group, the larval heart rate was significantly reduced in both the daytime (*t* (58) =4.166, *p* < 0.001) and nighttime (*t* (58) = 7.358, *p* < 0.001) noise exposure groups, with the nighttime noise group exhibiting a more pronounced decrease than the daytime noise group (*t* (58) = 2.94, *p* = 0.005; Figure 2E).

### 3.2. Activities of Antioxidant Enzymes and Expressions of Related Genes

**Figure 3:**
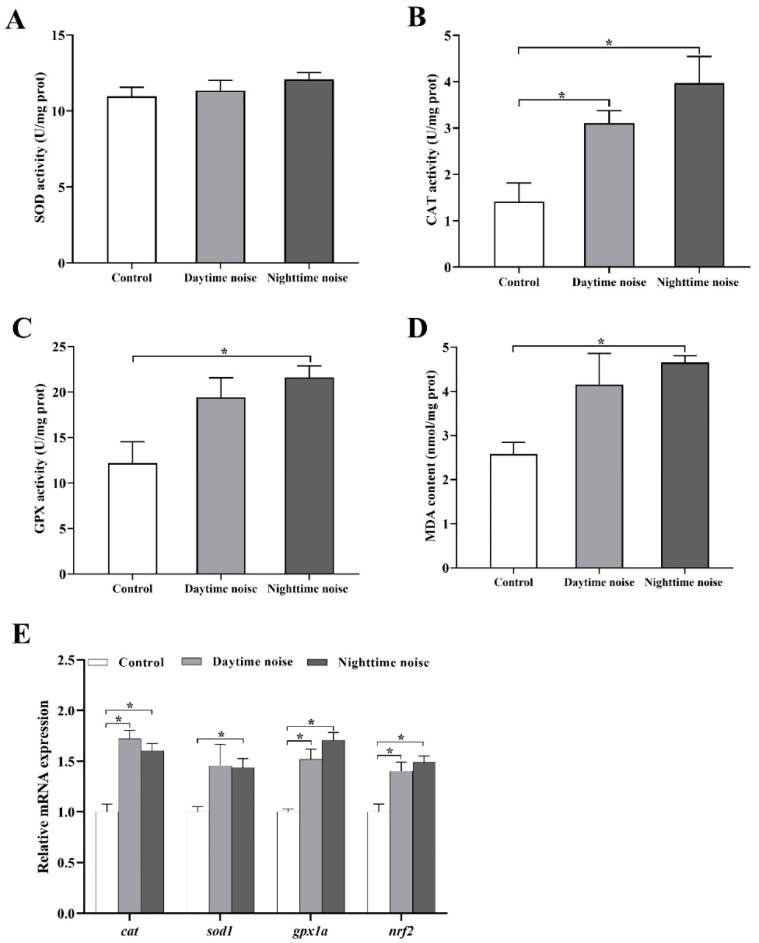
Oxidative stress responses in zebrafish larvae following noise exposure. (**A**) Superoxide dismutase (SOD) activity; (**B**) catalase (CAT) activity; (**C**) glutathione peroxidase (GPX) activity; (**D**) malondialdehyde (MDA) concentration; (**E**) relative mRNA expression of antioxidant-related genes. The data represent mean ± SEM (*n* = 3 replicates. Each replicate included approximately 200 larvae for (**A**–**D**), 20 larvae for (**E**)). The asterisks (*) denote significant differences between groups (*p* < 0.05); the comparisons between groups without asterisk annotations indicate no significant differences (*p* ≥ 0.05).

SOD activity showed no significant differences among groups (Figure 3A). Compared with the control group, CAT activity was significantly increased after exposure to both daytime (*t* (4) = −3.503, *p* = 0.044) and nighttime (*t* (4) = −3.644, *p* = 0.044) noise (Figure 3B), and the activity of GPX, as well as the content of MDA, increased following both daytime and nighttime noise exposure, with a statistically significant elevation observed in the nighttime noise group (GPX: *t* (4) = −3.534, *p* = 0.048; MDA: *t* (4) = −6.775, *p* = 0.004; Figure 3C,D).

Gene expression analysis revealed significant upregulation of *cat*, *gpx1a*, and *nrf2* in both daytime (*cat*: *t* (4) = −6.416, *p* = 0.006; *gpx1a*: *t* (4) = −5.049, *p* = 0.007; *nrf2*: *t* (4) = −3.497, *p* = 0.025) and nighttime (*cat*: *t* (4) = −5.933, *p* = 0.006; *gpx1a*: *t* (4) = −8.914, *p* = 0.002; *nrf2*: *t* (4) = −4.98, *p* = 0.016) noise-exposed groups (Figure 3E). Notably, *sod1* expression showed a significant increase only in the nighttime noise group (*t* (4) = −4.25, *p* = 0.044).

### 3.3. Otolith Development and Expressions of Related Genes

**Figure 4:**
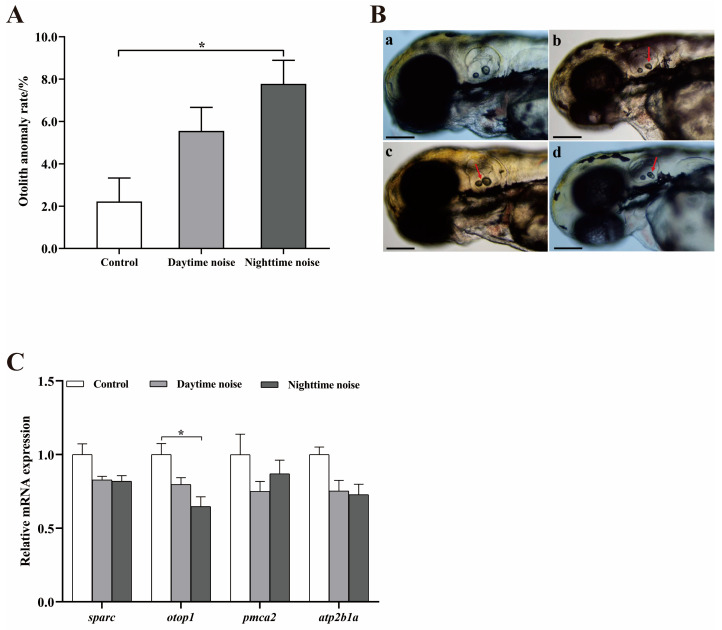
Otolith developmental abnormalities and gene expression changes. (**A**) Abnormal otolith rate; (**B**) representative images of otolith malformations (arrows; scale bar = 150 μm): (**a**) normal otoliths, (**b**) reduced area, (**c**) altered inter-otolith distance, (**d**) otolith division; (**C**) relative mRNA expression of otolith-related genes. The data represent mean ± SEM (*n* = 3 replicates; Each replicate included 30 larvae for (**A**,**B**), 20 larvae for (**C**)). The asterisks (*) denote significant differences between groups (*p* < 0.05); the comparisons between groups without asterisk annotations indicate no significant differences (*p* ≥ 0.05).

Both daytime and nighttime noise exposure increased the otolith developmental abnormality rate in larvae, with the nighttime group exhibiting a significantly higher rate than the control (*t* (178) = −3.542, *p* = 0.048; Figure 4A). The abnormalities mainly manifested as a reduction in otolith area, a change in the distance between the two otoliths, and otolith division (Figure 4B).

Gene expression analysis demonstrated significant downregulation of *otop1* in the nighttime noise group (*t* (4) = 3.533, *p* = 0.048; Figure 4C). Expression levels of *sparc*, *pmca2* and *atp2b1a* were only slightly downregulated, but the differences were not significant.

### 3.4. Number of Lateral Neuromasts and Expressions of Related Genes

**Figure 5:**
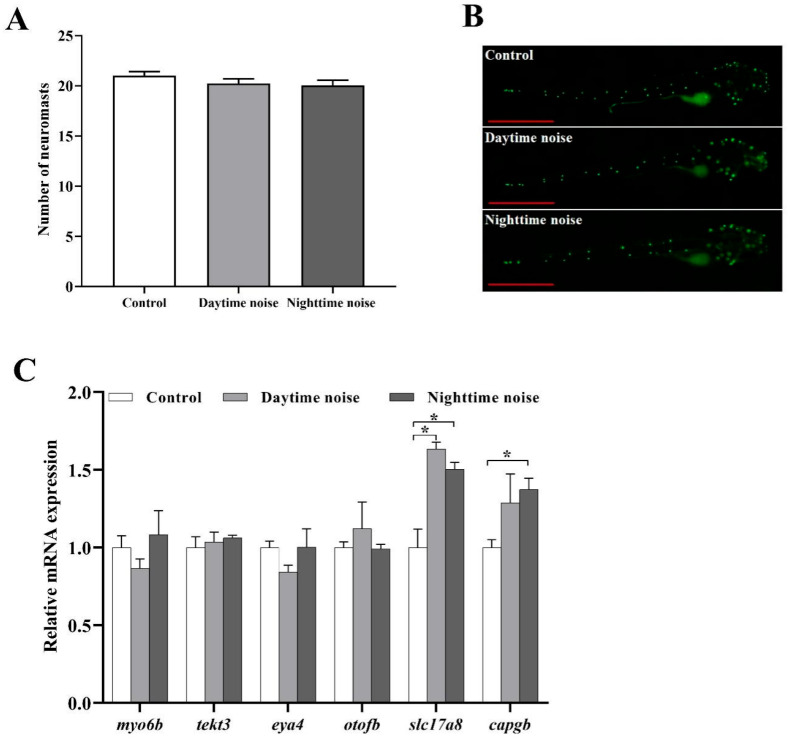
Lateral line neuromast analysis and hair cell gene expression. (**A**) Neuromast counts; (**B**) representative YO-PRO-1-stained neuromasts (green fluorescence; scale bar = 1 mm); (**C**) relative mRNA expression of hair cell-related genes. The data represent mean ± SEM (*n* = 30 for A; *n* = 3 replicates for (**C**), and each replicate included 20 larvae). The asterisks (*) denote significant differences between groups (*p* < 0.05); the comparisons between groups without asterisk annotations indicate no significant differences (*p* ≥ 0.05).

In both the daytime noise and nighttime noise groups, the number of lateral neuromasts exhibited a slight decrease; however, this change was not statistically significant (Figure 5A,B).

Regarding the expression of genes related to hair cell development, there were no significant differences in the expression levels of *myo6b*, *tekt3*, *eya4*, and *otofb* among the groups. Nevertheless, the expression level of *slc17a8* was significantly upregulated following exposure to both daytime (*t* (4) = −5.089, *p* = 0.014) and nighttime (*t* (4) = −4.03, *p* = 0.016) noise. Moreover, compared to the control group, the expression of *capgb* was also significantly increased in the nighttime noise group (*t* (4) = −4.258, *p* = 0.026; Figure 5C).

## 4. Discussion

The early growth and development stage is a critical period for the establishment of phenotypic traits in fish. Existing studies have demonstrated that noise can adversely affect the growth and development of fish embryos and larvae, leading to physiological stress and behavioral disorders [38,46,47,48,49]. The present study demonstrated that while daytime and nighttime noise exposure did not significantly alter the final cumulative hatching rate of zebrafish embryos by 120 h, both treatments induced a notable delay in the hatching process. Specifically, embryos exposed to noise exhibited significantly lower hatching rates at 48 h and 72 h compared to controls, with nighttime exposure exerting a more pronounced effect at 72 h. These findings suggested that noise pollution, particularly during nighttime, could disrupt the developmental timing of zebrafish embryos without ultimately reducing overall hatching success. The observed hatching delay may be attributed to altered energy allocation in noise-exposed embryos. The yolk sac serves as the primary nutrient source for embryos and early larvae. Noise exposure has been shown to accelerate yolk sac consumption while slowing growth [38]. The mechanical energy from underwater sound waves may cause excessive molecular vibration, inducing stress responses in developing embryos. Such stress could increase metabolic demands, leading to faster depletion of yolk sac reserves and diverting energy away from key developmental processes, ultimately delaying hatching [38,48].

Although noise exposure did not result in statistically significant increases in embryo or larval mortality, it is noteworthy that mortality rates in both daytime and nighttime noise-exposed groups were exceeded those in the control group, suggesting that noise stress may exert subtle but persistent negative effects on zebrafish embryonic viability. The trend was particularly pronounced in the nighttime exposure group, which maintained elevated cumulative mortality throughout the experimental period. While these differences did not reach statistical significance under our experimental conditions, the consistent trend indicated a biologically relevant impact that could become more pronounced under prolonged or higher-intensity exposure scenarios. This observation is supported by study on spiny chromis (*Acanthochromis polyacanthus*), where motorboat noise reduced embryo survival [49]. Heart rate is a vital indicator for evaluating heart activity and function. The significant reduction in larval heart rate observed in our study further supported the notion that noise disrupted cardiovascular development. Elevated plasma glucose levels, as reported in noise-exposed gilthead sea bream (*Sparus aurata*), may contribute to microcirculation disorders and myocardial ischemia, leading to decreased heart rate [9]. Furthermore, although the heart rate of zebrafish typically increases during early developmental stages [50], noise exposure group exhibited a decrease in heart rate, which may be attributed to the interference of noise stress on the developmental process. Specifically, accumulating evidence indicates that noise stress can lead to increased consumption of the yolk sac, resulting in stunted growth [36]. This physiological impairment could partially explain the slight increase in mortality observed in noise-exposed larvae. Additionally, the higher deformity rates observed in noise-exposed larvae, particularly at night, further underscore the detrimental effects of noise on embryonic development. Similar developmental abnormalities have been reported in scallop embryos exposed to noise [51], suggesting a common stress response across aquatic species.

Our results demonstrated that underwater noise exposure induced significant oxidative stress in developing zebrafish larvae, consistent with findings in Lusitanian toadfish (*Halobatrachus didactylus*), where boat noise (104–140 dB) disrupted the balance between oxidative stress and antioxidant defenses [47]. Environmental stressors like noise promote excessive free radical generation, activating protective antioxidant mechanisms. The SOD-CAT system serves as the first line of defense, with SOD converting superoxide radicals to H_2_O_2_, which CAT then detoxifies into water [52,53]. While Faria et al. [47] reported increased SOD activity in noise-exposed toadfish, we observed significantly elevated CAT activity in zebrafish larvae exposed to both daytime and nighttime noise, alongside unchanged SOD levels. This discrepancy may reflect either insufficient SOD upregulation or compensatory enhancement of alternative antioxidant pathways. GPX, which also breaks down H_2_O_2_ [54], exhibited increased activity in both noise-exposed groups, with nighttime exposure causing a more pronounced rise, further confirming noise-induced oxidative stress. Noise exposure led to elevated MDA levels, particularly at night, indicating lipid peroxidation (LPO) and membrane damage—a hallmark of oxidative stress that can trigger cell death [55]. These findings align with noise-induced GPX and MDA increases in rats [56]. At the molecular level, noise upregulated *sod1*, *cat*, and *gpx1a* expression, suggesting transcriptional control of antioxidant enzymes. Notably, *nrf2,* a key regulator of the Nrf2/Keap1-ARE pathway [57], was significantly upregulated in both noise-exposed groups, reflecting activation of this critical antioxidant response system. Together, these results demonstrated that noise exposure disrupted redox balance, prompting a multifaceted antioxidant defense involving enzymatic activity and gene expression changes.

The auditory system of fish comprises both the inner ear and lateral line system, with zebrafish larvae exhibiting acoustic responsiveness as early as 3 days post-fertilization [33,58]. The inner ear’s sensory macula is covered by paired calcium carbonate otoliths (utricle and saccule), which are critical for hearing and balance in fish [59]. In this study, noise exposure caused distinct otolith abnormalities, including reduced otolith area, altered inter-otolith spacing, and otolith fragmentation. Quantitative analysis revealed significantly higher otolith malformation rates in noise-exposed groups, particularly under nighttime exposure, suggesting noise disrupted normal otolith morphogenesis. The mechanistic basis for these defects may involve disrupted calcium carbonate and/or protein deposition. Otoliths are primarily composed of calcium carbonate, proteins, and a small fraction of fibrous material, with deficiencies in calcium carbonate deposition or constituent proteins leading to developmental abnormalities [60]. In our study, noise exposure downregulated *otop1*, the gene critical for otolith formation. *Otop1* encodes otopetrin 1, a protein involved in transporting otolith constituent proteins. Knockout of *otop1* disrupted otolith development, resulting in malformed, misplaced otoliths and a lack of organic matrix [61,62].

The lateral line system, another critical mechanosensory organ in fish, detects hydrodynamic stimuli through neuromasts containing hair cell clusters [63]. While neuromast counts showed no significant changes following noise exposure, the observed upregulation of *slc17a8* and *capgb*, two genes essential for hair cell development and auditory function [64,65], indicated that noise-induced hair cell damage triggered compensatory regeneration. Unlike mammals, zebrafish possess hair cell regenerative capacity [30], which likely accounted for the maintenance of stable neuromast numbers despite potential noise-induced damage.

The more pronounced effects of nighttime compared to daytime noise exposure observed in this study likely resulted from multiple interacting factors related to zebrafish biology and environmental interactions. As diurnal animals, zebrafish exhibit distinct activity-rest cycles, with nighttime representing a critical period for physiological recovery and developmental processes [35]. The disruption of this rest phase by noise may impair stress response mechanisms while simultaneously exacerbating oxidative damage and developmental abnormalities. The absence of natural daylight signals during nighttime exposure could further compromise protective pathways, including circadian-regulated antioxidant defenses, since oxidative stress responses in fish are known to be modulated by light–dark cycles [66]. The lack of visual stimuli at night may also increase reliance on acoustic perception, potentially increasing vulnerability to auditory system damage. Moreover, noise during this resting period appears to disrupt energy allocation, causing embryos to shift metabolic resources toward stress responses at the expense of normal developmental processes, which corresponds with our observations of elevated otolith abnormalities and deformity rates in larvae exposed to nighttime noise. These findings suggest that noise exposure during biologically critical nighttime periods disproportionately disrupts key developmental pathways, although future studies are needed to elucidate the precise mechanisms underlying this time-dependent sensitivity. Collectively, these results highlight the need for temporal considerations in noise pollution management, as nighttime acoustic disturbances might pose a particularly severe threat to aquatic organisms.

## 5. Conclusions

In summary, our study demonstrated that underwater noise, particularly during nighttime, adversely affected zebrafish embryonic development by delaying hatching, reducing heart rate, and increasing deformity rates. These effects were mediated through oxidative stress, as evidenced by elevated CAT and GPX activities, increased MDA levels, and the upregulation of antioxidant-related genes. Furthermore, noise disrupted otolith development and could damage hair cells in the lateral line system, with nighttime exposure causing more severe impairments. These findings underscore the importance of considering temporal patterns of noise pollution in aquatic environments and highlight the need for targeted mitigation strategies to protect early life stages of fish. Future research should investigate specific signaling pathways underlying noise-induced organ developmental defects and long-term consequences of noise-induced developmental disruptions.

## Figures and Tables

**Table 1:** Primers sequences used in this study.

Gene	Sequence (5′ to 3′)	Size (bp)	Accession NO.
*sparc*	F: GCAAGAAGGGCAAAGTGTGTGA R: AGAAGTGGCAGGAGGACTCGTA	147	AY575072.1
*otop1*	F: CTGCTGCTGGTGCTGGAGAAGT R: CCGTGGTTGAGGATGCCGTCAT	146	NM198803.1
*pmca2*	F: TCCGCCATTACCGTCATCATCC R: GCCACCACCAGCACTGTAACA	149	EF591990.1
*atp2b1a*	F: CATCCAGGGCAACGACCTCAAA R: CCGACAGCAGTGACCACGATT	147	HM449162.1
*myo6b*	F: CGGCGTTCTTCATCTCGGCAAT R: AGGCTCACTCTCAGGTCGTCCT	144	AY691328.1
*tekt3*	F: TGAGACGGCAGACACCAAGAAC R: CCAGACGGGTTTGGGCAACTT	146	XM696077.6
*eya4*	F: CACTCACTCCTCACTGGCTCCT R: GCACCTGGTCGCACTCCTCTAA	148	NM001282173.1
*otofb*	F: CCTCAACACAGCGTTCCAGACA R: TTCCACACTCCTCCTCCACAGA	118	XM021467487.1
*slc17a8*	F: CATGTCTTCGTGATCGCCTCCA R: CACCAATGATGCCGCACTTCTC	130	NM001082835.1
*capgb*	F: GGACTTGCTGGTGCGTGATGA R: CCTCTTCTCTTCTGCGTTGGCT	100	NM001001594.2
*cat*	F: TTGGAGCTTGCGTCCTGAATCG R: GTGTGCGATCCGTATCCGTTCA	102	NM130912.2
*sod1*	F: ATGTGACCGCTGATGCCAGTG R: TTTCCTCATTGCCACCCTTCCC	144	NM131294.1
*gpx1a*	F: GACGACCCTGTGTCCCTTATGG R: CGATGGTGAGGAACCTTCTGCT	148	NM001007281.2
*nrf2*	F: CTCCGCTCCACCTTCCACTGAT R: GCATTGGCATGTTGAGGCACTG	146	AB081314.1
*ef1α*	F: GATCACTGGTACTTCTCAGGCTGA R: GGTGAAAGCCAGGAGGGC	121	FJ915061
*β-actin*	F: CGAGCTGTCTTCCCATCCA R: TCACCAACGTAGCTGTCTTTCTG	86	AF025305.1

## Data Availability

Data is contained within the article.
